# Supplementary material for: Mucosal Antibody Response to SARS-CoV-2 in Paediatric and Adult Patients: A Longitudinal Study
Source: Pathogens. 2022 Mar 24;11(4):397. doi: 10.3390/pathogens11040397 (PMC9026526; doi:10.3390/pathogens11040397)
Supplement: Supplementary file 1 [file pathogens-11-00397-s001.zip › pathogens-1615565-supplementary.pdf]

Supplementary Material

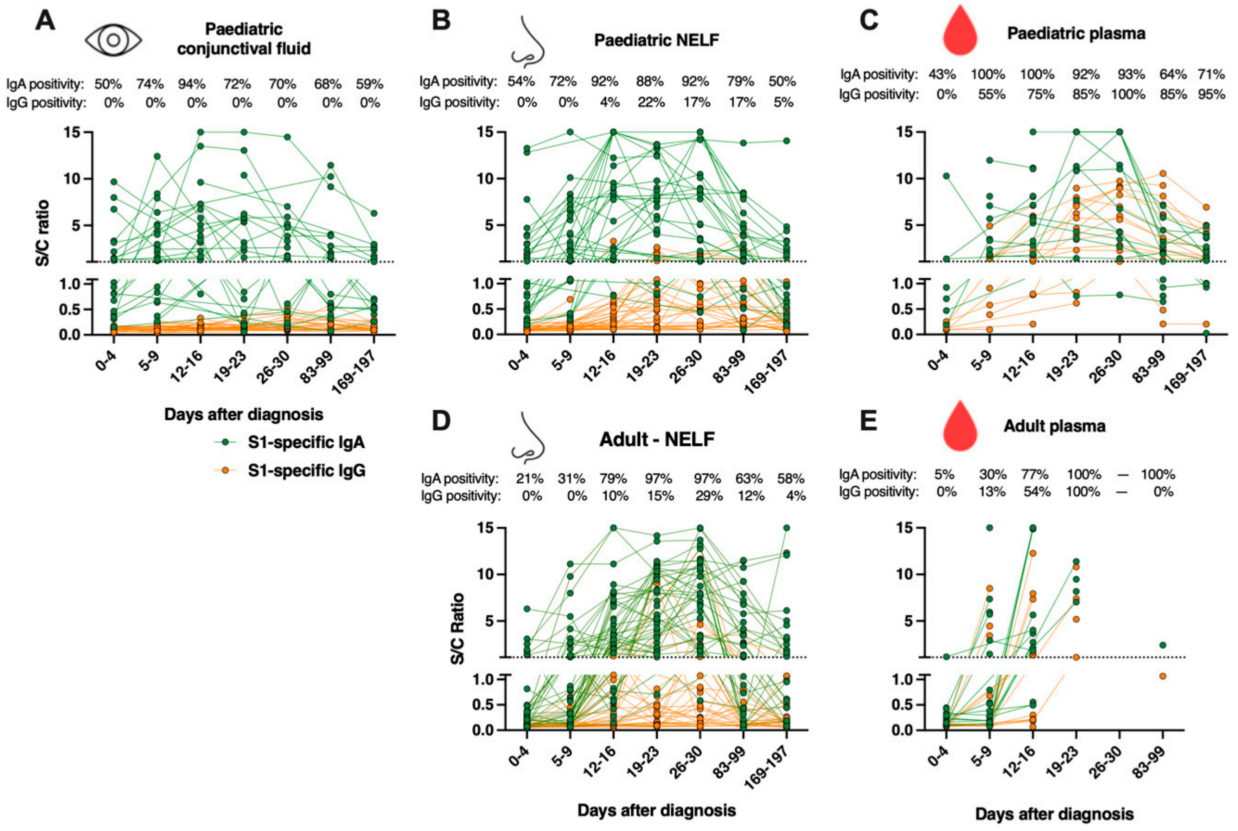

Supplementary Figure S1. SARS-CoV-2 S1-specific antibody levels in COVID-19 patients. The longitudinal changes of S1-specific IgA and IgG in the **(A)** conjunctival fluid, **(B)** nasal epithelial lining fluid (NELF) and **(C)** plasma of paediatric patients and the **(D)** NELF and **(E)** plasma of adult patients were plotted. Data points above the dotted line (Sample/Calibrator (S/C) ratio ≥ 1.1) are considered positive, while y=15 indicates the upper detection limit of the assay. The percentages denote the IgA and IgG positivity at each time point.

A. Conjunctival Fluid (CF) - IgA

Days post diagnosis		0-4	5-9	12-16	19-23	26-30	83-99	169-197
All Paediatric patients	No. of sample	20	22	17	18	20	19	17
	%	50	74	94	72	70	68	59
Asymptomatic	n	9	9	5	5	8	8	7
	Median ± IQR	0.9±6.8	2.1±3.2	2.3±2.4	0.7±4.0	1.0±1.9	1.1±2.3	0.6±1.4
	%	44	67	80	40	50	50	43
Symptomatic	n	11	13	12	13	12	11	10
	Median ± IQR	1.3±1.8	1.9±7.1	5.4±6.3	5.6±7.0	5.0±4.6	2.8±5.5	2.1±3.1
	%	55	77	100	85	83	82	70
Mann-Whitney test between groups	*p* value	0.8820	0.5556	**0.0365**	0.1433	**0.0252**	0.1087	**0.0431**
Fisher's Exact test between groups	*p value*	ns	0.6550	0.2941	0.0987	0.1611	0.3189	0.3500

B. Nasal epithelial lining fluid (NELF) - IgA

Days post diagnosis		0-4	5-9	12-16	19-23	26-30	83-99	169-197
Paediatric patients	No. of sample	28	29	25	25	24	24	22
	%	54	72	92	88	92	79	50
Asymptomatic	n	11	10	6	7	7	8	7
	Median ± IQR	2.9±6.3	5.8±6.1	9.3±13.0	8.7±8.7	7.1±9.3	2.2±4.9	0.7±2.1
	%	82	90	83	86	86	63	43
Symptomatic	n	17	19	19	18	17	16	15
	Median ± IQR	0.7±.13	1.5±3.1	8.2±12.7	7.7±8.8	8.3±10.7	3.1±5.6	1.8±2.4
	%	35	63	95	89	94	88	53
Mann-Whitney test between groups	*p* value	**0.0017**	0.0854	0.7778	0.9400	0.4641	0.4167	0.1417
Fisher's Exact test between groups	*p* value	**0.0238**	0.2008	0.4300	ns	0.5072	0.2885	ns

C. NELF - IgG

Days post diagnosis		0-4	5-9	12-16	19-23	26-30	83-99	169-197
Paediatric patients	No. of sample	26	26	24	23	24	24	22
	%	0	0	4	22	17	17	5
Asymptomatic	n	10	8	6	5	7	8	7
	Median ± IQR	0.1±0.1	0.2±0.1	0.5±0.9	0.6±0.3	0.2±0.4	0.4±0.5	0.1±0.1
	%	0	0	17	0	0	13	0
Symptomatic	n	16	18	18	18	17	16	15
	Median ± IQR	0.1±0.0	0.1±0.1	0.2±0.2	0.5±1.2	0.6±1.0	0.4±0.8	0.2±0.3
	%	0	0	0	28	24	19	7
Mann-Whitney test between groups	*p* value	0.1956	**0.0293**	0.1008	0.8006	**0.0389**	0.4614	**0.0319**
Fisher's Exact test between groups	*p* value	ns	ns	0.2500	0.5453	0.2833	ns	ns

D. Plasma - IgA

Days post diagnosis		0-4	5-9	12-16	19-23	26-30	83-99	169-197
Paediatric patients	No. of sample	7	11	12	13	13	14	14
	%	43	100	100	92	92	64	71
Asymptomatic	n	1	7	3	1	2	4	4
	Median ± IQR	0.200	6.8±6.2	11.0±5.4	6.800	2.8±2.9	0.8±1.0	1.0±2.8
	%	0	100	100	100	100	25	25
Symptomatic	n	6	4	9	12	11	10	10
	Median ± IQR	1.1±1.4	2.9±3.6	5.2±5.3	10.9±11.2	11.0±11.5	2.5±3.7	1.9±2.8
	%	50	100	100	92	91	80	90
Mann-Whitney test between groups	*p* value	-	0.2303	0.1455	-	0.2821	0.0759	0.1419
Fisher's Exact test between groups	*p* value	-	ns	ns	-	ns	0.0949	**0.0410**

E. Plamsa - IgG

Days post diagnosis		0-4	5-9	12-16	19-23	26-30	83-99	169-197
Paediatric patients	No. of sample	7	11	12	13	13	13	14
	%	0	55	75	85	100	85	86
Asymptomatic	n	1	7	3	1	2	3	4
	Median ± IQR	0.100	1.7±4.5	7.1±5.7	2.800	2.2±1.4	2.0±4.9	1.8±1.0
	%	0	57	100	100	100	100	75
Symptomatic	n	6	4	9	12	11	10	10
	Median ± IQR	0.1±0.1	1.0±1.1	1.3±3.0	5.2±4.7	7.0±4.8	4.8±6.6	2.3±3.2
	%	0	50	67	83	100	80	90
Mann-Whitney test between groups	*p* value	-	0.4121	**0.0364**	-	0.1538	0.4685	0.3037
Fisher's Exact test between groups	*p* value	-	ns	0.5091	-	ns	ns	0.5055

Table S1. Comparisons of the SARS-CoV-2 S1-specific antibody levels between asymptomatic vs symptomatic paediatric patients with mild disease. The antibody levels in S/C ratios at the same time point were compared by the Mann-Whitney test, while the percentages of positive samples were compared by Fisher's Exact test. *P* values smaller than 0.05 are bolded, *p* values >0.9999 are represented by ns (not significant) while dashes mean no data for comparison.

A. Nasal epithelial lining fluid (NELF)

**Days post diagnosis**	**IgA**	**0-4**	**5-9**	**12-16**	**19-23**	**26-30**	**83-99**	**169-197**
Adult patients	No. of sample	32	42	43	35	35	30	23
	n	13	17	18	15	14	10	8
Mild disease	Median ± IQR	0.3±1.7	1.1±4.0	3.4±5.5	4.7±6.7	5.9±8.5	1.1±4.2	1.7±3.9
	%	31	47	89	93	93	50	63
	n	13	17	17	13	12	11	8
Moderate disease	Median ± IQR	0.3±0.4	0.6±0.9	2.8±5.4	7.2±7.1	9.3±6.1	4.9±10.4	2.8±11.2
	%	14	28	67	100	100	73	67
	n	6	8	8	7	9	9	7
Severe & critically ill	Median ± IQR	0.2±0.6	0.2±1.0	3.5±6.5	8.4±5.1	9.8±5.7	2.6±5.5	9.5±2.4
	%	17	25	75	100	100	67	43
Dunn's multiple comparisons test	*p-values*							
	Adult patients: mild disease vs moderate disease	0.9469	0.4095	0.7753	0.1974	0.6076	0.3648	ns
	Adult patients: mild disease vs severe & critically ill	0.3959	0.1715	ns	0.3957	0.8992	ns	ns
	Adult patients: mild disease vs severe & critically ill	ns	ns	ns	ns	ns	ns	ns
Fisher's Exact test between groups	*p-values*							
	Adult patients: mild disease vs moderate disease	0.6447	0.2818	0.2285	ns	ns	0.3870	ns
	Adult patients: mild disease vs severe & critically ill	ns	0.4018	0.5633	ns	ns	0.6499	0.6193
	Adult patients: mild disease vs severe & critically ill	ns	ns	ns	ns	ns	ns	0.6193

**Days post diagnosis**	**IgG**	**0-4**	**5-9**	**12-16**	**19-23**	**26-30**	**83-99**	**169-197**
Adult patients	No. of sample	26	30	29	27	28	26	23
	n	10	10	10	10	9	8	7
Mild disease	Median ± IQR	0.1±0.1	0.1±0.1	0.1±1.0	0.2±0.2	0.6±0.8	0.1±0.3	0.1±0.1
	%	0	0	20	0	11	0	0
	n	10	12	11	10	10	9	9
Moderate disease	Median ± IQR	0.1±0.0	0.1±0.1	0.1±0.2	0.2±1.0	0.4±2.5	0.2±0.4	0.1±0.2
	%	0	0	0	22	40	13	0
	n	6	8	8	7	9	9	7
Severe & critically ill	Median ± IQR	0.1±0.1	0.1±0.02	0.3±0.8	0.5±2.8	0.5±3.1	0.7±0.9	0.2±0.9
	%	0	0	13	29	33	22	14
Dunn's multiple comparisons test	*p-values*							
	Adult patients: mild disease vs moderate disease	ns	ns	ns	0.931	ns	ns	ns
	Adult patients: mild disease vs severe & critically ill	0.0751	**0.0249**	ns	**0.0221**	ns	ns	0.1921
	Adult patients: mild disease vs severe & critically ill	0.3243	**0.0461**	ns	0.2357	ns	ns	0.2809
Fisher's Exact test between groups	*p-values*							
	Adult patients: mild disease vs moderate disease	ns	ns	0.2143	0.4737	0.3034	ns	ns
	Adult patients: mild disease vs severe & critically ill	ns	ns	ns	0.1544	0.5765	0.4706	ns
	Adult patients: mild disease vs severe & critically ill	ns	ns	0.4211	ns	ns	ns	0.4375

B. Plasma

**Days post diagnosis**	**IgA**	**0-4**	**5-9**	**12-16**	**19-23**	**26-30**	**83-99**	**169-197**
Adult patients	No. of sample	19	23	13	4	0	1	0
	n	7	8	4	1	0	1	0
Mild disease	Median ± IQR	0.2±0.3	2.2±4.9	2.9±7.3	8.2	-	2.4	-
	%	14	63	75	100	-	100	-
	n	10	9	4	3	-	-	-
Moderate disease	Median ± IQR	0.2±0.3	0.3±3.0	2.9±4.2	9.5±4.3	-	-	-
	%	0	22	75	100	-	-	-
	n	2	6	5	-	-	-	-
Severe & critically ill	Median ± IQR	0.2±0.1	0.2±0.2	14.9±13.4	-	-	-	-
	%	0	0	80	-	-	-	-
Dunn's multiple comparisons test	*p-values*							
	Adult patients: mild disease vs moderate disease	ns	0.2657	ns	-	-	-	-
	Adult patients: mild disease vs severe & critically ill	ns	**0.0476**	0.7271	-	-	-	-
	Adult patients: mild disease vs severe & critically ill	ns	ns	0.8495	-	-	-	-
Fisher's Exact test between groups	*p-values*							
	Adult patients: mild disease vs moderate disease	0.4118	0.1534	ns	ns	-	-	-
	Adult patients: mild disease vs severe & critically ill	ns	**0.0310**	ns	-	-	-	-
	Adult patients: mild disease vs severe & critically ill	ns	0.4857	ns	-	-	-	-

**Days post diagnosis**	**IgG**	**0-4**	**5-9**	**12-16**	**19-23**	**26-30**	**83-99**	**169-197**
Adult patients	No. of sample	19	23	13	4	0	1	0
	n	7	8	4	1	0	1	0
Mild disease	Median ± IQR	0.1±0.0	0.3±0.7	1.5±1.8	10.8	-	1.1	-
	%	0	13	75	100	-	0	-
	n	10	9	4	3	-	-	-
Moderate disease	Median ± IQR	0.1±0.0	0.1±1.7	0.2±1.0	5.2±6.3	-	-	-
	%	0	22	25	100	-	-	-
	n	2	6	5	-	-	-	-
Severe & critically ill	Median ± IQR	0.1±0.0	0.1±0.0	7.3±9.9	-	-	-	-
	%	0	0	60	-	-	-	-
Dunn's multiple comparisons test	*p-values*							
	Adult patients: mild disease vs moderate disease	0.3689	0.6938	0.4390	-	-	-	-
	Adult patients: mild disease vs severe & critically ill	0.9629	ns	ns	-	-	-	-
	Adult patients: mild disease vs severe & critically ill	ns	ns	0.1334	-	-	-	-
Fisher's Exact test between groups	*p-values*							
	Adult patients: mild disease vs moderate disease	ns	ns	0.4857	ns	-	-	-
	Adult patients: mild disease vs severe & critically ill	ns	ns	ns	-	-	-	-
	Adult patients: mild disease vs severe & critically ill	ns	ns	0.5238	-	-	-	-

Table S2. Comparisons of the SARS-CoV-2 S1-specific antibody levels between adult patients of different disease severity. The antibody levels in S/C ratios at the same time point were compared by the Kruskal-Wallis test followed by Dunn's multiple comparison, while the percentages of positive samples were compared by Fisher’s Exact test. P values smaller than 0.05 are bolded, p values >0.9999 are represented by ns (not significant) while dashes mean no data for compa

	Paediatrics	Adult all	Adult 1	Adult 2	Adult 3+4
	n = 20	n = 43	n = 17	n = 15	n = 11
Day between symptom onset and diagnosis (median ± IQR)	0 ± 1	-1 ± 3	-1 ± 2	-2 ± 2	-2 ± 3

Table S3: The median and interquartile range of the period between onset of symptoms and day of diagnosis. Negative median means the symptoms had started before the PCR positive laboratory-based diagnosis.

Days post diagnosis	**Paediatric patients**				**Adult patients**					
	Asymptomatic		Symptomatic		Mild disease		Moderate disease		Severe/critically ill	
	n	Median ± IQR	n	Median ± IQR	n	Median ± IQR	n	Median ± IQR	n	Median ± IQR
**0**	8	34.0±6.2	11	18.3±5.6	4	17.7±4.1	5	18.7±14.5	3	21.7±9.0
**1**	7	34.0±5.1	8	21.5±14.6	5	22.6±10.2	4	22.4±10.4	1	20.2±0
**2**	0	-	5	20.7±4.5	6	17.8±8.9	1	24.1±0	2	23.6±16.4
**3**	9	31.4±10.6	3	33.2±6.4	3	27.9±10.7	9	18.2±9.0	2	22.3±5.2
**4**	5	35.0±6.1	3	31.1±18.1	3	23.8±11.1	5	22.1±8.9	2	24.3±15.5
**5**	9	35.0±7.1	10	30.5±7.4	3	27.2±11.7	6	23.2±12.7	3	35.0±15.8
**6**	5	35.4±10.0	6	31.1±8.0	4	29.3±6.8	4	26.1±9.5	6	21.4±8.5
**7**	3	40.0±7.1	9	32.7±11.2	7	27.0±18.4	1	23.0±0	2	18.7±1.6
**8**	2	33.8±12.3	8	37.5±9.4	7	35.0±16.3	8	28.3±11.2	6	26.3±7.7
**9**	4	40±0	9	35.0±9.4	4	30.4±17.4	4	35.0±1.4	1	24.4±0
**10**	4	40±3.8	6	30.0±6.2	2	37.5±5.0	4	33.9±8.3	3	30.0±9.8
**11**	1	35.0±0	4	35.05±5.2	5	31.46±8.6	3	32.6±6.1	3	30.6±9.5
**12**	2	35.0±10.1	7	31.8±5.1	1	40±0	3	30.9±9.3	2	26.9±3.5
**13**	1	40±0	2	35.0±0	3	35.0±18.3	5	35.0±11.3	3	23.7±12.4
**14**	-	-	5	35.0±3.1	4	34.2±6.0	4	34.8±10.1	1	40±0
**15**	2	37.5±5.0	4	37.5±5.0	3	34.2±17.0	2	37.3±5.4	4	29.4±6.4
**16**	1	35±0	6	35.0±5.6	1	35.0±0	3	25.6±2.5	2	25.8±4.3
**17**	-	-	2	36.3±7.4	1	31.1±0	-	-	1	22.9±0
**18**	-	-	2	30.1±6.4	-	-	2	36.1±7.8	3	29.7±1.5
**19**	-	-	1	40±0	1	37.6±0	1	40±0	3	27.5±7.0
**20**	-	-	-	-	1	33.7±0	1	27.9±0	2	31.4±3.6
**21**	-	-	-	-	-	-	0		3	30.9±14.8
**22**	-	-	-	-	-	-	0		2	36.2±7.7
**23**	-	-	-	-	-	-	1	27.4±0	1	27.9±0
**24**	-	-	-	-	-	-	0		1	29.2±0
**25**	-	-	-	-	-	-	1	40±0	1	29.5±0
**26**	-	-	-	-	-	-	1	35.0±0	1	30.0±0

Table S4: The number of specimen (n) and the median and interquartile range (IQR) of the CT values of the corresponding patient group on each day.

Patients	Comorbidities
1	Hypertension (HT), Hx of cerebrovascular accident, Bipolar Affective Disorder, Alcoholism
2	Diabetes mellitus (DM), HT, Obese, Gout (Year 2010), Microalbuminuria, known obstructive sleep apnea (OSA) not on continuous positive airway pressure (CPAP)
3	Benign prostatic hyperplasia (BPH)
4	Hyperlipidaemia and Hypothyroidism
5	Atrial fibrillation with long pause
6	Non-ulcer dyspepsia, Helicobacter pylori eradication, Renal stone extracorporeal shock wave lithotripsy (ESWL) & surgery
7	DM, ruptured anterior communicating artery (ACoA) aneurysm with craniotomy and clipping done, pneumococcal meningitis in year 2015

Table S5: The comorbidities of the seven adult patients with severe COVID-19.
